# Nursing environment and delirium in ICU patients

**DOI:** 10.1186/cc9754

**Published:** 2011-03-11

**Authors:** IJ Zaal, LM Peelen, CF Spruyt, J Kesecioglu, AJ Slooter

**Affiliations:** 1 University Medical Centre Utrecht, the Netherlands

## Introduction

Delirium is a common and serious disorder in the ICU. It has been suggested that the ICU environment may play a role in the development of ICU delirium, but this has never been investigated. In this study we aimed to investigate the relationship between the nursing environment and the duration, incidence and severity of ICU delirium.

## Methods

This prospective observational before/after study was performed in the 32-bed, mixed adult ICU of the University Medical Centre Utrecht. All patients admitted to the ICU were daily assessed on delirium by research physicians. Exclusion occurred when patients remained unresponsive (RASS <-3) during admission or when they were unable to understand Dutch and English. ICU delirium was compared between a ward-like setting, and a setting with single-patient, noise-reduced rooms with diurnal light variation.

## Results

A total of 55 patients (449 observations) were included in the old setting and 75 patients (468 observations) in the new setting. Demographic characteristics were similar for both groups. However, co-morbidity was more severe and emergency admissions were more frequent in the new setting. Delirium occurred in 28 (51%) patients in the old setting versus 34 (45%) patients in the new setting (*P *= 0.53). After adjusting for confounding, the days patients spent in delirious state decreased with 0.4 days in the new environment (*P *= 0.005). No difference could be observed in the severity of delirium or in the medications prescribed.

## Conclusions

The number of days patients spent delirious during ICU admission was found to be shorter in patients who were treated in separate noise-reduced rooms with diurnal light variation despite a similar incidence and severity of ICU delirium.

